# Knowledge and Attitudes towards Prostate Cancer and Screening among Males in Limpopo Province, South Africa

**DOI:** 10.3390/ijerph20065220

**Published:** 2023-03-22

**Authors:** Ndivhuwo Maladze, Angelina Maphula, Mzamani Maluleke, Lufuno Makhado

**Affiliations:** 1 Department of Psychology, Faculty of Health Sciences, University of Venda, Private Bag X5050, Thohoyandou 0950, Limpopo, South Africa; 2 Department of English Media Studies and Linguistics, Faculty of Humanities, Social Sciences and Education, University of Venda, Private Bag X5050, Thohoyandou 0950, Limpopo, South Africa; 3 Department of Public Health, Faculty of Health Sciences, University of Venda, Private Bag X5050, Thohoyandou 0950, Limpopo, South Africa

**Keywords:** prostate cancer, health, attitudes, knowledge, screening, South Africa, Limpopo

## Abstract

Inadequate knowledge and poor attitudes about prostate cancer (PC) negatively affect early screening practices among males. The PC mortality rate is increasing due to late reporting, screening, and treatment. This study explored the awareness, attitudes, and PC screening behaviours among males in the Limpopo, Thulamela municipality. This descriptive cross-sectional study involved 245 males that were randomly selected. A structured questionnaire was used to collect data. Fisher’s exact tests and logistic regression analysis were used to examine the association between sociodemographic variables, awareness, and attitudes towards PC. Our findings revealed that 64.1% demonstrated inadequate awareness about PC. The overall score (84.9%) showed a positive attitude towards PC. However, 87.4% had a negative attitude towards the effectiveness of treatment for PC. The majority (96.7%) of respondents had never undergone a PSA test, although 53.1% were willing to undergo a PSA test. There was a significant positive correlation between awareness of prostate cancer and attitudes toward prostate cancer (r = 0.280, *p* < 0.001). Health status predicted awareness about PC, while age and health status predicted attitudes towards PC among men. Rural community-based programmes and heightened awareness campaigns are needed to conscientize men about the risk factors, symptoms, diagnosis, and treatment of PC in rural areas of Limpopo.

## 1. Introduction

Prostate cancer (PC) is the malignancy that affects most adult males globally and has a devastating impact if not discovered early. It is currently regarded as the second most diagnosed type of cancer and contributes to the increasing death rate in adult males [1,2]. Studies revealed a high prevalence of PC among older men because they have moderate knowledge about the symptoms, while some have a negative attitude towards early screening [3,4,5,6,7]. The leading causes of PC are not clearly outlined, but age is the most common risk factor, where the highest incidences have been found in males aged 65 and above [8,9]. In 2018, global statistics for PC incidence and mortality rate were 1,276,106 and 358,989, respectively [10], whereas in 2020, they rose to 1,414,259 incidences and 375,304 mortalities [11]. These statistics confirm that the rate of infection is accelerating, and tentative measures should be implemented.

Numerous studies conducted indicate that high percentages of affected males are in developed countries, followed by developing countries, which can be attributed to inadequate knowledge about PC [12,13,14]. Developed countries have sufficient resources to conduct awareness campaigns and encourage males to be screened in their early forties, which is not the case in developing and underdeveloped countries. A study conducted in Nigeria among males older than 40 years indicated that only 47.5% of the respondents knew about PC [15]. In another study conducted in Saudi Arabia among men over 40 years, it was found that only 10% of the 400 respondents practised regular PC examination check-ups because of little knowledge about the disease [16].

In Africa, South Africa has many PC cases, and the rate seems to be growing faster [17]. This may have been due to the fact that white males in SA, though in the minority, present themselves for screening earlier and have access to improved prostate-specific antigen (PSA) testing and diagnostic facilities compared to black males [18]. Most black South African males have limited PC knowledge, and they are diagnosed when it has reached an advanced and aggressive stage [19]. A total of 54.4% of the respondents in one study conducted in South Africa indicated that they had never heard about PC [20]. This paints a gloomy picture regarding black males’ knowledge of PC and some measures to improve their knowledge.

Major contributory factors which can be associated with a high rate of PC malignancy and lack of screening include cultural beliefs, lack of knowledge, health beliefs, lack of adequate surgical care, as well the use of traditional medicine to cure unusual ailments [4,21,22,23]. Additionally, black males have a negative attitude towards screening, resulting in severe pain when discovered late and a high mortality rate [24,25]. Respondents in a study conducted in Kenya regarded PC as a myth as they had misconceptions that it is associated with sexual behaviours [26]. However, some researchers argued that there is still controversy when using prostate-specific antigen (PSA) testing for screening because it has possible negative health consequences [27,28,29].

Providing adequate knowledge to males who are less aware of screening would ignite interest in registering, thus improving their health [30]. It can also inspire males who get tested early to spread the information even to deep rural areas where they have never heard about PC. Literature reveals few studies conducted in South Africa on the awareness, attitudes, and screening practices towards PC among males, especially in rural areas. Therefore, this study focuses on the awareness, attitude, and PC screening behaviours of males in Limpopo Province, South Africa, to fill the gap and advocate for measures to encourage awareness, early screening, and treatment of PC.

## 2. Materials and Methods

### 2.1. Study Design and Sampling

This study followed a quantitative cross-sectional design involving males aged 40 years and above from Dzingahe Village in the Thulamela Municipality, Limpopo Province. During a community gathering, the headman/chief recruited 570 males from Dzingahe village to participate in the study. The study sample frame was composed of all males aged 40 years and above who registered their names and contact details. The sample size was determined using Raosoft because of its great strength, exceptionally robust reliability, and its proven system that possesses high data integrity [31]. Two hundred and thirty (230) was an appropriate sample, and the researcher added 15 in case of default or errors; 245 respondents were randomly sampled through simple random sampling. The procedure for simple random sampling involved assigning numbers to all participants and placing them in a container. The numbers were mixed to ensure that each one had an equal chance of being chosen. The desired sample size was achieved by manually selecting participants based on their assigned numbers, and if any selected participant declined to participate, they were replaced with another randomly selected individual. In this study, approximately 15 males declined to participate. Data were collected in August of 2018.

### 2.2. Data Tools and Data Collection

A structured self-administered questionnaire was used that contained questions organized in four sections. Section A: sociodemographic information with seven items; Section B: prostate cancer awareness with eleven items; Section C: attitude towards prostate cancer screening with eight items on a 4-point Likert scale; and Section D: PC screening behaviours with seven items. The questionnaire used was adapted from a study conducted on the knowledge, attitudes, and screening practices regarding prostatic diseases among Nigerian men [14]. The sociodemographic information focused on the individual characteristics of the sampled men, such as age, marital status, educational level and occupation, income, religion, reported health status, and reasons for poor health. The awareness domain consisted of 11 multiple choice questions (MCQs) to measure respondents’ awareness regarding prostate cancer. For each correctly answered question, the participant was scored ‘1’ and for the incorrectly answered questions, ‘0’. To ease the comparison, the awareness status was divided into inadequate and adequate awareness based on the scores obtained by each respondent. Out of the maximum score of eleven, each respondent who scored five or less was categorized as having inadequate awareness. Each respondent who obtained six or more was categorized as having adequate PC awareness.

To assess the attitudes regarding PC screening, the researcher used eight statements on a 4-point Likert scale: strongly agree, agree, disagree, and strongly disagree. The scale was scored as strongly agree, ‘1’; agree, ‘1’; disagree, ‘0’; and strongly disagree, ‘0’ for all the positive questions and strongly agree, ‘0’; agree, ‘0’; disagree, ‘1’; and strongly disagree, ‘1’ for all the negative questions. Out of the maximum score of eight, each respondent who scored four or more was classified as having a positive attitude. Each participant who scored three or less was classified as having a negative attitude towards screening for prostate cancer. The style of the Likert scale was adapted from [16]. Back-translation to the original version (English) was performed by language experts to ensure the conceptual and cultural correspondents of the two versions the quality and accuracy of the instrument.

To gain access to the headman/chief, the researchers were assisted by the community civic structure and traditional council. A Tshivhidzo meeting (community gathering) was held at the headman’s place (Musanda) to share information about the purpose and processes of the project to all community males who met the required criteria. Data were collected in a group setting, and the date and time for data collection were arranged with respondents together with the leaders of the community (civic structure and traditional council).

### 2.3. Data Management and Analysis

The collected data were coded and entered twice independently on Microsoft Excel then later exported and analysed using the Statistical Package for Social Science (SPSS) version 26.0 (IBM SPSS, Chicago, FL, USA) [32]. Descriptive statistics were used to summarize the data. Inferential statistics were utilized to test for associations and effect size, respectively, at a 0.05 level of significance. The odds ratio was used to assess the likelihood of reporting awareness and attitudes for two-by-two tables. Awareness was an explanatory variable in this analysis, while attitudes and screening practices were response variables. The demographic variables included age, education, marital status, socioeconomic status, education, and occupation. The Pearson correlation coefficient (*r*) was used to establish the association between sociodemographic variables, awareness, and attitudes towards PC. Direct logistic regression was performed to assess the impact of demographic characteristics on the likelihood that respondents would report being aware of PC and their attitudes towards PC. The model contained seven (7) independent variables (age, religion, marital status, employment status, income level, level of education, and health status). The use of the Pearson correlation coefficient and logistic regression in this study may have been motivated by the desire to explore the relationships between variables, model the impact of demographic characteristics on awareness/attitudes towards PC, and control for potential confounding variables.

## 3. Results

A total of 245 respondents participated in this study. Table 1 represents the demographic profile of respondents; 70.3% were aged 60 years or below, 54.5% were married, more than half of the respondents (61.4%) were employed/self-employed, 11% had no formal education, while 55.3% had the post-matric qualification, and 26.4% followed tradition. About 12.2% admitted that their health is poor, and 8.2% suffered from chronic illness.

Table 2 represents the respondents’ awareness of PC; 62.4% had no prior awareness about prostate cancer, only 12.2% learnt about PC from a physician, 60.8% could identify what PC is, and 35.1% of respondents identified family history as a risk factor. About 69% of respondents were not aware of the age at risk for the development of prostate cancer, 81.2% reported that they had no prior knowledge about PSA and DRE screening methods, 51.8% believed that prostate cancer could be treated, 35.9% did not know, while 12.2% said it could not be treated. Most of the respondents (64.1%) had inadequate awareness about PC in general and its available screening services.

Table 3 represents respondents’ attitudes towards prostate cancer screening. More than half of all men (67.8%) positively responded towards adults undergoing PC screening, 82.8% regarded early consultation with doctors regarding urinary symptoms to be helpful, 87.4% had a negative attitude towards the effectiveness of treatment for PC, about 67.8% had a negative attitude towards screening for PC if a person is healthy and fit, while 60.8% reported that they would only consider going for PC screening when sick/ill. The overall score (84.9%) showed a positive attitude towards PC.

Table 4 represents men’s PC screening practices; 96.7% of respondents have never in their life undergone a PSA test, 88.2% have never consulted a physician concerning prostatic problems, and 53.1% were willing to undergo a PSA test. Among those who consulted their physicians regarding prostatic problems (11.8%), 7.3% had consulted only once. The reasons reported for not consulting were (25.3%) financial constraints, 18.4% did not see any PC symptoms, 13.1% felt that they were not at risk, and 12.2% were not aware of PC screening. A total of 18.4% of respondents would undergo a PSA screening test to know their status, 17.2% would not undergo a PSA screening test because they do not feel like they are at risk to have PC, 10.6% thought they did not need PSA screening because they were not sick, and 7.3% lacked interest to undergo a PSA screening test.

There was a significant positive correlation between awareness of prostate cancer and the attitudes toward prostate cancer (r = 0.280, *p* < 0.001). A significant negative association was marked between the awareness of prostate cancer and age (r = −0.352, *p* = 0.001). A significant negative association was also marked between attitude towards prostates cancer and marital status (r = 0.194, *p* = 0.002) and (r = 0.183, *p* = 0.004), respectively. Thus, most married respondents show positive attitudes compared to those who were separated, divorced, widowed, or single. Awareness was positively associated with employment status, the monthly minimum poverty line income, level of education, and health status at (r = −0.421, *p* = 0.001; r = −0.455, *p* = 0.001; r = 0.346, *p* = 0.001; and r = 0.488, *p* = 0.001). Furthermore, attitudes toward prostate cancer were also shown to be positively correlated with the level of education (r = 0.258, *p* < 0.001) (see Table 5).

The full model containing all predictors was statistically significant, x^2^ (7, N = 245) = 20.64, *p* = 0.004, indicating that the model could distinguish between respondents who reported and those who did not report awareness of prostate cancer. The model explained between 29.8% (Cox and Snell R square) and 40.7% (Nagelkerke R squared) of the variance in awareness of prostate cancer and accurately classified 80.0% of cases. As shown in Table 6, only one (1) of the independent variables made a unique statistically significant contribution to the model (health status). The findings revealed the odds ratio of 0.20 for good health status, which was less than one, indicating that respondents who possessed good health status were 0.20 times less likely to report being aware of prostate cancer. This was also followed by those with poor health status who were 0.05 times less likely to report prostate cancer awareness.

The full model containing all predictors was statistically significant, x^2^ (7, N = 245) = 16.18, *p* < 0.04, indicating that the model could distinguish between respondents who reported positive and those who reported negative attitudes towards prostate cancer. The model explained between 22.6% (Cox and Snell R square) and 39.5% (Nagelkerke R square) of the variance in attitudes towards prostate cancer and accurately classified 89.0% of cases. As shown in Table 7, only three (3) independent variables made a unique statistically significant contribution to the model (age and health). The findings revealed the odd ratio of 0.05 and 0.16 for age and health, respectively, which were less than one, indicating that respondents aged 61 years and above as well as those with poor health were 0.05 and 0.16 times less likely to report negative attitudes toward prostate cancer, respectively.

## 4. Discussion

This study was carried out to explore males’ awareness, attitude, and PC screening behaviours in Limpopo Province, South Africa. In our study, more than half of the participants had not heard of and reported inadequate awareness about PC. Similar findings were also found in a study conducted in Muldersdrift among patients attending a Urology clinic where more than half (54.4%) had never heard about PC, and 90.2% of respondents never knew of the existence of prostate cancer [33]. By contrast, some African countries found that more than three quarters, 94.9%, had a high level of knowledge about prostate cancer and 54.1% were aware of PC, respectively [34,35]. Poor awareness was also reported in some other studies [14,36]. There is still a lot to educate the public about regarding prostate cancer in Limpopo Province, given the low reported awareness about the disease, especially in rural areas.

Interestingly, in our study, family, friends, and physicians were the sources of information. Similar findings have been observed in Italy, where physicians, family, and friends were informants. On the contrary, television and newspapers were identified as sources [3,14]. The physicians/primary health care providers have an essential role to play in educating the public about the screening and treatment services for prostate cancer to achieve early diagnosis and successful treatment for PC.

In our study, less than half of the respondents (35.1%) were able to identify some risk factors for the development of prostate cancer. Similar findings were reported in a study carried out in South Africa, where about 32.3% of respondents were aware that family history is a risk factor for prostate cancer development [22]. On the contrary, in developed countries, high rates of respondents reported a high level of knowledge of risk factors [3]. Men’s awareness about the age at risk for the development of prostate cancer was low, as three quarters of respondents did not know; and that cancer can be present without symptoms at all. On the contrary, more than half (65.9%) of respondents were aware of the age at risk for developing PC [3]. Similar findings were observed in a study conducted among men in Ghana, where 69.6% of the respondents reported that they were not aware that prostate cancer was an asymptomatic disease [37]. This might be one of the reasons why most men are diagnosed with metastatic stage cancer (when it has spread to other parts of the body). This shows how important it is for strategies to be put into place to raise awareness about PC to the public if early detection and treatment is to be achieved. Thus, the gap in awareness and knowledge about PC among men is a concern as it impedes screening behaviours. The knowledge regarding screening services for prostate cancer was relatively low, as only less than a quarter of respondents reported having prior knowledge about PSA (prostate-specific antigen) testing, and as high as (64.1%) of respondents had no idea that PSA and DRE testing are used to detect prostate cancer. These findings correspond to a study in SA where 76% of respondents were unable to identify any screening service for prostate cancer [36], and in Nigeria, only a quarter (25.1%) had heard about PSA [14].

On the contrary, a high knowledge about prostate cancer screening services was reported in Italy [3]. The difference might be influenced by the huge difference in health care facilities. Despite being unaware of screening services for PC, more than half of respondents (53.1%) showed interest and willingness to undergo PSA examination. Dissimilar with other findings, most respondents were willing to be screened for PC [14,38]. A little less than half of the men (46.9%) reported that they would never go for PC screening. This is concerning because PC screening provides early detection and better treatment outcomes. These findings are comparable with a study conducted in Spain where 42.1% of respondents were not willing to undergo a PC screening test [39]. In contrast, the study by Ojewala et al. [14] reported the lowest rate (5.6%) of men who were unwilling to undergo the screening.

In our study, more than three quarters of respondents (84.9%) demonstrated positive attitudes towards the PC. Above 67.8% of men showed positive attitudes towards undergoing PC screening. These findings were a little lower when compared to the study conducted among men in Namibia, where 91.1% showed a willingness to be screened for PC [38]; Ugandan men reported poor attitudes towards the PC screening [7]. These differences might be attributed to the low levels of awareness about PC and access to screening services. Although men reported positive attitudes towards PC screening, only 11.8% had consulted a physician. Similar findings were reported in Nigeria, whereby only a few (less than one fifth) respondents had been screened for prostate cancer [37]. The current results are slightly different from a study conducted in SA in which only one out of 182 men had been screened for PC [33]. Poor PC screening rates among men might be connected to the fact that the primary health care in rural communities might not readily have screening services available. To attain a sustainable, effective control and treatment of this cancer, the health care sector and other NGOs should incorporate and strengthen their services to focus more on raising awareness about PC to the public and providing screening services in all primary health care facilities.

Reasons for not screening were being healthy, not being sick, and being fit, and more than half of the men had a negative attitude regarding the effectiveness of drug treatment. Men reported that consulting a physician is only necessary when one is sick and when home remedy fails. In this regard, the Health Belief Model (HBM) and Stages of Change Theory can provide insights into the reasons for non-screening among men for prostate cancer. According to the HBM, individuals are more likely to engage in a health behaviour if they perceive themselves as susceptible to the condition, if the condition has serious consequences, and if the benefits of the behaviour outweigh the costs [40]. In this case, men may not be screening for prostate cancer because they perceive themselves as healthy, fit, and not at risk for the disease. They may also have negative attitudes towards the effectiveness of drug treatment, which can reduce their motivation to engage in screening.

The Stages of Change Theory proposes that individuals move through a series of stages when changing their behaviour, including precontemplation, contemplation, preparation, action, and maintenance [41]. Men who are in the precontemplation stage may not be considering screening at all, while those in the contemplation stage may be weighing the pros and cons of screening. Men who are in the preparation stage may be actively seeking information and resources to help them engage in screening, while those in the action and maintenance stages have already started screening and are working to maintain the behaviour.

Based on these theories, strategies to promote prostate cancer screening could include increasing awareness of the risk of prostate cancer and the benefits of screening, addressing negative attitudes towards drug treatment, providing information and resources to men who are in the contemplation or preparation stages, encouraging men to consult with a physician even if they feel healthy, and providing screening services in convenient and accessible locations [40,41].

In a study by Wong et al. [42], men reported that consulting a physician is only necessary when one is sick and when home remedy fails [42]. This finding highlights the importance of addressing attitudes towards preventive care and promoting the benefits of screening even for seemingly healthy individuals. Overall, using the HBM and Stages of Change Theory can help to inform targeted interventions to promote prostate cancer screening among men who may be reluctant to engage in the behaviour.

Similar findings were reported in the study conducted in Namibia, where men had undergone PC screening because they were worried and felt sick [38]. In contrast, in a study conducted among Italian men, slightly more than half had been screened following a physician’s recommendation [3]. Additionally, financial constraints, having no symptoms, and perceiving oneself to be at no risk of developing PC were cited as reasons for not screening. Consulting when other remedies fail defeats the purpose and predisposes men to prostate cancer spreading before it is diagnosed, leading to a high mortality rate. Physicians in developed countries seem to have screening services readily available. Still, in low-income settings, physicians are burdened with patient load operating in under-resourced facilities making it difficult to refer for PC screening. Thus, physicians have a role in influencing men to undergo PC screening, provided that the health facilities have the necessary equipment and personnel.

An association was noted between awareness of PC and attitudes toward PC. Old age was negatively associated with awareness towards PC, while level of education, employment status, monthly poverty line income, religion, and health status were positively associated with awareness. Attitudes toward PC were also shown to be positively correlated to education. This is corroborated by several other studies in Africa [33,37,43,44]. On the contrary, no association was found in [14]. Those who are educated have better jobs and income, might have more profound insight, and can afford consultations with physicians compared to unemployed men.

Most married men reported positive attitudes as compared to the ones who were separated, divorced, widowed, or single. This is corroborated by a study done in SA [22]. In our study, health status was a predictor of awareness of PC; men with poor health were more likely to report awareness, while those who reported good health were less likely to report awareness of PC. Age and health status predicted attitudes towards PC; men aged 61 years and above as well as those with poor health were less likely to report negative attitudes toward PC. Older men are more exposed to prostate cancer-related problems. Awareness is an indicator for men seeking knowledge and possibly changing attitudes towards PC screening, prevention, and management and thus solely depends upon early screening. In our study, men reported poor screening behaviours, which is a risk as it prohibits early management of potential cancer cases among men.

On the other hand, health facilities in rural areas of Limpopo might not be fully equipped to readily screen for PC. Our study calls for more awareness campaigns and programmes in rural communities to heighten awareness about PC and screening services. The government and policymakers might want to incorporate a mandatory PC screening into the primary health care system for men above 40 years of age to curb late PC diagnosis. In our study, the media did not play a role in PC awareness. Therefore, it is recommended that the government’s intervention should target media platforms to ensure that PC is aired on radios, television, and any other media channel to reach men in rural communities. PC screening services must be made accessible and affordable. Future research may focus on the barrier to early PC screening.

Furthermore, sources [45,46,47,48,49] suggest that population/cohort screening in time intervals can be an effective approach to detecting prostate cancer early and improving outcomes and that NGOs and universities can play a valuable role in increasing awareness and facilitating access to screening services. However, there is ongoing debate about the benefits and risks of prostate cancer screening, and screening recommendations may vary depending on individual factors such as age, family history, and overall health.

There is a need for a prevention program that includes population or cohort screening in time intervals and involves the use of NGOs or universities as sources of information and services. This could be a possible option to facilitate access to prostate cancer screening services.

NGOs and universities can provide outreach and education to the public about the importance of prostate cancer screening and help to increase awareness and understanding of the screening process. They can also help to coordinate and provide screening services in a community setting, which can be more convenient and accessible for individuals who may not have easy access to healthcare facilities.

Additionally, a prevention program that includes population or cohort screening can help to identify individuals who may be at higher risk for prostate cancer and allow for early detection and treatment, which can improve health outcomes and reduce mortality. This type of program can also help to reduce disparities in access to screening services, particularly for underserved populations.

However, it is important to note that the implementation of such a program would require careful planning and coordination, including developing appropriate screening guidelines, training healthcare providers, and ensuring adequate resources and funding for the program. It is also important to consider potential drawbacks or limitations, such as the possibility of overdiagnosis or unnecessary treatment and the need to ensure that screening is conducted in an ethical and equitable manner.

### Limitations

The study has limitations as it showed a high odds ratio (OR) value, which suggests that the effect size may have been overestimated. It is possible that confounding variables were not adequately controlled for despite using the Pearson correlation and logistic regression techniques, which could have contributed to the high OR. It is important to note that the study’s findings may not be applicable to other regions, but they can serve as a basis for future research aimed at enhancing awareness and uptake of PC screening.

## 5. Conclusions

The increase in PC morbidity and mortality calls for studies to explore men’s awareness, attitudes, and PC screening behaviours. Although men reported positive attitudes, our respondents’ lack of awareness and poor PC screening behaviour raises a deep concern regarding PC control, early detection, diagnosis, and effective management among men in rural communities in Limpopo Province. Demographic variables were significantly associated with men’s PC awareness and attitudes. Health status predicted awareness about PC, while age and health status predicted attitudes towards PC among men. Rural community-based programmes and heightened awareness campaigns are needed to conscientize men about the risk factors, symptoms, diagnosis, and treatment of this deadly disease in rural areas of Limpopo.

## Figures and Tables

**Table 1:** Demographic profile of males (n245) in Dzingahe village.

Variable	Frequency (n)	Percentage (%)
Age:		
≤60 years	173	70.3%
>61 years	72	29.3%
Marital status:		
Married	134	54.5%
Single/Widowed	56	22.8%
Separated/Divorced	55	22.4%
Employment status:		
Unemployed/Pensioner	94	38.2%
Employed/Self-Employed	151	61.4%
Income: < & >Minimum/Poverty line		
<R3501	117	47.6%
>R3501	128	52%
Educational status:		
None	27	11%
Primary level/Secondary level	82	33.3%
Diploma (TVET)/University	132	55.3%
Religion:		
Catholic/Protestant	80	32.5%
Traditional	65	26.4%
Muslim	100	40.7%
Reported health status		
Excellent	95	38.8%
Good	72	29.4%
Fair	48	19.6%
Poor	30	12.2%
Reasons for poor health:		
Chronic illness	20	8.2%
Acute illness	3	1.2%
Disability	7	2.9%

**Table 2:** Assessment of men’s awareness regarding prostate cancer in Dzingahe village.

Awareness Statements	Frequency (n)	Percentage (%)
Have you ever heard about prostate cancer?		
No	153	62.4%
Yes	92	37.6%
Source of information		
Physician	30	12.2%
Mass media	20	8.2%
Internet	6	2.4%
Friend/Family	36	14.7%
What do you think prostate cancer is?		
Cancer of the male reproductive organ	37	15.1%
Cancer of the prostate gland	149	60.8%
Don’t know	59	24.1%
Possible risk factors for the development of prostate cancer		
Family history	86	35.1%
Alcohol	26	10.6%
High-fat diet	42	17.1%
Older age	67	27.3%
Smoking	21	8.6%
Obesity	3	1.2%
Gender mostly affected by prostate cancer		
Men only	175	71.4%
Women only	5	2%
Both men and women	43	17.6%
Don’t know	22	9%
Age at risk for prostate cancer		
Yes	76	31%
No	169	69%
Signs for prostate cancer		
Fever	10	4.1%
Loss of appetite	37	15.1%
Blood in urine	73	29.8%
Pain during urination	51	20.8%
Loss of weight	30	12.2%
Headache	6	2.4%
Frequent urination	38	15.5%
Ever heard of the PSA and DRE?		
No	199	81.2%
Yes	46	18.8%
Source of Information		
Internet	4	8.9%
Physician	23	51.1%
Friends/Family	18	40.0%
Mass media	0	0
Others	0	0
PSA and DRE are used to detect prostate cancer		
No	11	4.5%
Yes	77	31.4%
I don’t know	157	64.1%
Do you think prostate cancer can be treated?		
Yes	127	51.8%
No	30	12.2%
I don’t know	88	35.9%
Level of awareness		
Adequate awareness	88	35.9%
Inadequate awareness	157	64.1%

**Table 3:** Men’s attitude towards prostate cancer in Dzingahe village.

Statements	Positive Attitude n (%)	Negative Attitude n (%)
All adults should undergo prostate cancer screening.	166 (67.8%)	79 (32.3%)
Early diagnosis of prostate cancer improves clinical outcome.	218 (88.6%)	27 (11.4%)
Early consultation with doctors for urinary symptoms is helpful.	203 (82.8%)	42 (17.2%)
Drug treatment of prostatic diseases is effective.	30 (12.2%)	215 (87.4%)
Medical and surgical treatment can cure prostatic problems.	225 (91.5%)	20 (8.1%)
Consultation with a doctor is only necessary when a home remedy fails.	129 (52.7%)	116 (47.3%)
Screening for prostate cancer is not necessary if one is healthy and fit.	167 (67.8%)	79 (32.2%)
I will only consider prostate cancer screening when I get sick/ill.	149 (60.6%)	96 (39.2%)
Attitude levels towards prostate cancer		
Positive attitude	208	84.9%
Negative attitude	37	15.2%

**Table 4:** Men’s screening practices for prostate cancer in Dzingahe village.

Screening Practices Questions	Frequency	Percentage
Have you ever consulted a physician regarding prostate cancer?		
Yes	29	11.8%
No	216	88.2%
Have you ever undergone a PSA test?		
Yes	8	3.3%
No	237	96.7%
Would you undergo a PSA test?		
Yes	130	53.1%
No	115	46.9%
How frequently have you consulted your physicians about prostatic related problems?		
Once	19	7.8%
Twice	8	3.3%
More than three times	2	0.8%
None	216	88.2%
Reasons why you have undergone a PSA screening test?		
I felt sick	4	1.6%
I felt at risk	4	1.6%
Reasons why you have never undergone a PSA screening test?		
Financial constraints	62	25.3%
I see no reasons since I have no symptoms	45	18.4%
I don’t feel at risk	32	13.1%
Unaware of the screening	30	12.2%
It’s a rare disease in our area/country	21	8.6%
Lack of interest	18	7.3%
Never advised by the physician	16	6.5%
It’s a rare disease in our people	13	5.3%
Reasons why you would want to do a PSA screening examination?		
To detect cancer before symptoms occur	61	27.3%
To know my status	45	18.4%
If I am sick	12	4.9%
If I know PSA screening	6	2.4%
Reasons why you would never have PSA screening done?		
I don’t feel at risk	47	17.2%
I don’t feel sick	26	10.6%
Lack of interest	18	7.3%
It’s a rare disease	18	7.3%
Lack of time	6	2.4%

a Some answers allowed respondents to tick more than one answer. b Some respondents did not have to answer certain questions if they would not undergo PC testing.

**Table 5:** Correlations of demographic variables and men’s awareness and attitude towards prostate cancer in Dzingahe village.

	Awareness r (*p*-Value)	Attitudes r (*p*-Value)
Awareness of PC		0.280 (0.000) **
Age	−0.352 (0.000) **	−0.047 (0.465)
Marital status	0.007(0.915)	−0.194 (0.002) **
Employment status	0.421 (0.000) **	−0.183 (0.004) **
Monthly poverty line income	0.455 (0.000) **	0.099 (0.123)
Level of education	0.346 (0.000)	0.258 (0.000) **
Religion	−0.222 (0.000) **	−0.080 (0.213)
Health status	0.488 (0.000) **	−0.011 (0.859)

** *p*-value < 0.001.

**Table 6:** Logistic regression predicting awareness of prostate cancer.

Logistic Regression Predicting Awareness of Prostate Cancer								
	B	S.E.	Wald	df	Sig.	OR	95% C.I. for EXP(B)	
							L	U
Age (1)	0.313	1.027	0.093	1	0.761	0.73	0.098	5.469
Religion			2.414	2	0.299			
Religion (1)	0.612	0.402	2.316	1	0.128	0.82	0.839	4.052
Religion (2)	0.415	0.462	0.807	1	0.369	0.54	0.612	3.743
Marital status			1.303	2	0.521			
Marital (1)	−0.260	0.433	0.360	1	0.549	0.64	0.330	1.802
Marital (2)	0.703	0.618	1.292	1	0.246	1.29	0.147	1.663
Employment status (1)	−0.067	1.026	0.004	1	0.948	0.94	0.125	6.980
Income (1)	−1.186	0.764	2.412	1	0.120	3.27	0.068	1.365
Level of education			0.981	2	0.612			
Education (1)	−0.509	0.976	0.272	1	0.602	2.31	0.089	4.071
Education (2)	0.325	0.647	0.253	1	0.615	1.66	0.389	4.924
Health status			20.915	3	0.000			
Health (1)	−3.000	1.230	5.943	1	0.015	0.20	0.004	0.555
Health (2)	−1.101	0.765	2.072	1	0.150	0.33	0.074	1.489
Health (3)	−1.592	0.384	17.184	1	0.000	0.203	0.096	0.432
Constant	−0.508	1.233	0.170	1	0.680	1.662		

Variable(s) entered on step 1: Age, Religion, Marital, Occupational, Income, Education, Health Status.

**Table 7:** Logistic regression predicting attitudes towards prostate cancer.

Variables in the Equation								
	B	S.E.	Wald	df	Sig.	OR	95% C.I. for OR	
							L	U
Age (1)	−3.039	1.171	6.730	1	0.009	0.048	0.005	0.476
Religion			3.271	2	0.195			
Religion (1)	−0.430	0.530	0.660	1	0.417	0.650	0.230	1.837
Religion (2)	1.455	1.028	2.003	1	0.157	4.283	0.571	32.122
Marital			1.345	2	0.510			
Marital (1)	0.474	0.672	0.499	1	0.480	1.607	0.431	5.996
Marital (2)	−0.307	0.899	0.12	1	0.732	0.735	0.126	4.283
Employ. Status (1)	−22.02	8170.394	0.00	1	0.998	0.000	0.000	
Income (1)	18.71	8170.394	0.000	1	0.998	133,977,955.63	0.000	
Education			2.604	2	0.272			
Education (1)	−0.74	0.963	0.591	1	0.442	0.477	0.072	3.150
Education (2)	0.47	0.984	0.231	1	0.631	1.605	0.233	11.051
Health			10.043	3	0.018			
Health (1)	−0.71	1.147	0.379	1	0.538	2.027	0.214	19.186
Health (2)	−0.84	1.185	0.497	1	0.481	0.434	0.043	4.423
Health (3)	−1.78	0.622	8.147	1	0.004	0.169	0.050	0.573
Constant	5.79	1.610	12.943	1	0.000	328.079		

B—regression coefficient or slope coefficient; S.E.—standard error; Wald—Wald statistic; df—degrees of freedom; Sig.—significance level; OR—odds ratio; C.I.—confidence interval; L—lower bound; U—upper bound. (1) and (1)—categories of the binary dependent variable.

## Data Availability

The data presented in this study are available on request from the corresponding author. The data are not publicly available due to the nature of ethical clearance approved that did not include data sharing unless it is within the manuscript.
